# Impact of a global leader on pharmaceutical practice and policy around the world

**DOI:** 10.1186/s40545-020-00253-z

**Published:** 2020-08-21

**Authors:** F. Alves da Costa, M. Henman, C. Hughes, H. Leufkens, Z. Babar, J. McElnay, M. Schulz

**Affiliations:** 1 Faculty of Pharmacy, University of Lisbon, Lisbon, Portugal; 2 University Institute Egas Moniz, Lisbon, Portugal; 3 School of Pharmacy and Pharmaceutical Sciences, University of Dublin Trinity College, Dublin, Ireland; 4 Clinical and Practice Research Group, School of Pharmacy, Medical Biology Centre, Queen’s University Belfast, Belfast, UK; 5 Division of Pharmacoepidemiology and Clinical Pharmacology, Utrecht Institute for Pharmaceutical Sciences, Utrecht University, Utrecht, the Netherlands; 6 Pharmaceutical Policy and Practice Research Centre at the Department of Pharmacy, University of Huddersfield, Queensgate, Huddersfield, HD1 3DH UK; 7 Department of Medicine, ABDA - Federal Union of German Associations of Pharmacists, Berlin, Germany; 8 Institute of Pharmacy, Freie Universität Berlin, Berlin, Germany

**Keywords:** Pharmaceutical care, Medication review, Clinical pharmacy, Europe

## Abstract

This commentary describes the contributions of a Dutch pharmacist who contributed in a unique manner to the development of community pharmacy practice in Europe, to the evolution of practice-based research and to its publication. With an interest in pharmaceutical care and in clinical pharmacy, Dr. van Mil changed practice and policy in Europe over the last decades in a very visible way, here documented through a summary of some of his main written contributions. We write this to honour his memory and contribute to the preservation of his legacy.

The first reference to pharmaceutical care dates to 1975 [1], and successive definitions and operationalisation of the term have continued across the world. However, many of these were largely descriptive or pilot studies, with little impact on practice or simply refinements of terminology which did not transfer into policy which is essential to lead to changes in the scope of practice of pharmacists working in various health care settings.

A pragmatic approach, a strong character and a drive to make the world a better place are some of the phrases that could be used to describe Dr. Foppe van Mil, who died on 18 July 2020. This commentary summarises the most relevant achievements of a unique career that has had an impact on the practice, research or policy relating to pharmacy across the world, either working on his own or, more often, collaboratively with academic or practice-based teams. Table 1 summarises many of the projects with which Dr. van Mil was associated alongside some key publications that influenced practitioners, researchers and policy-makers, including his PhD thesis which was a landmark for pharmacy practice in the Netherlands, expanding its impact to many other countries [2].

**Table 1:** Summary of practice, research projects and recognitions that have impacted pharmaceutical care practice or policy

Title	Brief description	Countries involved	Impact	References
“Therapeutic Outcomes Monitoring” (TOM) project	TOM is a model for increasing pharmacists’ role in primary health care, based on a continuous quality improvement system applied to PhC to detect, prevent and resolve DRPs in asthma patients. This project was conducted as a controlled intervention study (grouped at the pharmacy level) and various outcomes were measured to assess the impact of the intervention (e.g. HRQoL, PEFR, satisfaction).	Austria, Belgium, Canada, Denmark, Florida (USA), Germany, Iceland, Northern Ireland, The Netherlands	These controlled intervention studies had a huge impact on implementing pharmacy practice research at the university level in many countries. Furthermore, results achieved led to recognise pharmacist in DMPs and clinical practice guidelines. Today, there are specific programmes, often remunerated, for pharmacists to support inhaled medication used, namely in Belgium, in Denmark, to name a few.	[2–5]
Elderly Medication Analysis (OMA project)	Study of the effect of PhC in the elderly (> 65 years) using 4 or more different drugs and living independently. Designed as a controlled study with randomisation at the pharmacy level and involved 1290 intervention patients (selected in 104 pharmacies) and 1164 controls (selected from 86 pharmacies). Outcomes measured included HRQoL, patient satisfaction and knowledge, provider satisfaction (GP and pharmacist), drug use (including adherence) and use of health services.	Sweden, Portugal, Northern Ireland, Denmark, Germany, Republic of Ireland, The Netherlands	This was a landmark study as it was the first multicentred study in European community pharmacies. It was influential in the field of pharmacy practice research.	[2, 3, 6]
Review article	Discusses the concept of PhC care from a European perspective and clarifies the status of PhC research and implementation. It discusses if PhC can be part of the practice of pharmacy.	Europe	It suggests changes in the pharmacy curriculum so that pharmacists can acquire new knowledge and skills. It encourages countries to standardise procedures adopted to guarantee every patient receives PhC.	[7]
Review article	Describes European developments in the implementation of and research into PhC, focusing on the community pharmacy.	Europe	Paper by invitation of the Harvard Health Policy Review (USA).	[8]
Opinion paper	Establishes the need and the rationale for using a classification of DRPs to avoid negative consequences for patients.	Germany	Motivated the adoption of such classifications in pharmacy practice in both primary and secondary care.	[9]
Terminology papers	This sequence of publications establishes the foundations for understanding concepts of PhC, clinical pharmacy and medication review, highlighting the boundaries and similarities between concepts, while reflecting the evolution in practices.	Worldwide, with a special focus in Europe	These definitions are used by academics and researchers worldwide to support teaching and educational activities.	[10–13]
Research paper	Development of a PCNE-DRP classification.	World	Currently being used in Belgium, China, Germany, Ghana, Norway, Poland, Portugal, Serbia, Slovenia, Spain, Sweden, and Taiwan.	[14–16]
Review paper	Describes the healthcare system context, pharmacy practice and range of services offered in pharmacies in the Netherlands.	The Netherlands	Used widely as reference to frame various research or practice based studies undertaken in this counrty	[17]
Review paper	Describes the healthcare system context, pharmacy practice and range of services offered in pharmacy in Peru.	Peru (South America)	Used widely as reference to frame various research or practice based studies undertaken in this counrty	[18]
Research paper	Describes the healthcare system context, pharmacy practice and range of services offered in pharmacy in Europe.	Europe	Provides a framework for countries to benchmark themselves against each other.	[19]
Research paper	Describes the medication review practices in Europe, implementation and remuneration models associated.	Europe	Provides an assessment of the implementation of medication review in Europe, enabling benchmark and analysis of the strong points of some of the countries described in more detail.	[20]
Editor-in-Chief of the Int J Clin Pharm	Published high-quality manuscripts that enhanced the visibility of pharmaceutical care and clinical pharmacy in various countries of the world.	Worldwide	These publications have provided evidence-based practice to motivate policy changes in many countries, including in some cases changes in the scope of practice or remuneration models for pharmacy.	Pharmacy practice commentary on social media (22 July 2020)

He was involved in the first two pharmaceutical care research projects in Europe; in the Asthma Intervention Project (TOM), he was a postgraduate student, and in the Care of the Elderly study (OMA), he was a leading member of the research team [3, 6]. Both projects took a holistic approach to patient care and involved international collaboration, two aspects that were characteristic of Foppe’s conception of research. The major influence on Foppe’s research involvement, and on his influence on his colleagues, was his experience and expertise as a community pharmacist; this made him very practical but also very demanding, because for him, research was about, and for, the benefit of patients. His reputation reached all countries, and even countries recognised as having an advanced practice, including the UK, benefited from visiting his practice in the Netherlands [21].

Foppe had a very visible contribution to the dissemination of research and pharmacy practice, by his various attributes and activities. He was an educator, but a practical one, who once committed, would work tirelessly to deliver high quality. He was involved for many years in the Programme Committee for the Continuing Education Programme organised by the Community Pharmacy Section of the International Pharmaceutical Federation (FIP), through which annual sessions were held as pre-satellite meetings. These sessions aimed at the continuous professional development of pharmacists, particularly in the area of pharmaceutical care, and have led to the improvement of knowledge and practice skills of community pharmacists [22]. The aims, development and impact measurements of this programme were published subsequently [23]. FIP and the World Health Organization (WHO) collaborated to deliver a course on pharmaceutical care in Uruguay and other Latin American countries and chose Foppe to do this. He used a train-the-trainer model, in which theory-based sessions were followed by visits to the settings where the trainers practised to better understand their reality and adapt the learnings to their needs.

Two of the main vehicles for the advancement and dissemination of knowledge in the field of pharmaceutical care in Europe are the European Society of Clinical Pharmacy (ESCP) and the Pharmaceutical Care Network Europe (PCNE). As a member of ESCP, Foppe delivered workshops and lectures over many years and helped to promote research through his contributions to the Communication Committee. His unique contribution was co-founding the PCNE (in 1994), and he was truly its backbone; irrespective of the challenges, whether organisational, fiscal or philosophical, Foppe persevered and his belief in the value of PCNE and consequently sustained everyone in it. The definition of pharmaceutical care, the classification of drug-related problems and the conception of medication reviews all depended on Foppe’s initiative, determination and implementation. In addition through national organisations, such as the “Förderinitiative Pharmazeutische Betreuung” (Foundation Pharmaceutical Care) in Germany and at national pharmacy conferences, for example, in Poland [24] and at special occasions such as the award ceremony of the Royal Pharmaceutical Society of Great Britain, Foppe was an invited and valued contributor.

Foppe van Mil was an extraordinary person in the Dutch pharmacy practice space and beyond. Always vocal, always committed to innovation for the well-being of patients and public health. He was a very straightforward, yet unorthodox person, who combined in a very original and appealing manner practice and academic work, at the University of Groningen. His drive to make a difference, both in teaching and in research, was amazing and has always been well acknowledged. He was a recipient of the Innovation Prize of the Royal Dutch Association of Pharmacists (KNMP), a unique signature of excellence and leadership. This public recognition of innovative practice with visible benefits for patients contributed to further dissemination of the concept of pharmaceutical care and its implementation in practice there [2]. But many other institutions and organisations have publicly recognised him for his contribution leading to the advancements in research and practice. In Spain, during the first International Congress of Pharmaceutical Care (Atención Farmacéutica, San Sebastian 1999), he received together with Doug Hepler and Linda Strand (both from the USA) the Pharmaceutical Association of Gipuzkoa Award, as the judges considered these three individuals were, at that time, those with the most significant contributions to the advancement of pharmaceutical care internationally and whom, as such, stimulated the move to further the concept of pharmaceutical care in Spain, leading to its recognition and establishment in the law some years later.

In addition to Foppe’s contribution to the dissemination of research and practice innovation to researchers and pharmacy practitioners through his lectures and workshops, he made a very significant contribution in knowledge translation through his efficient and effective editorship of the International Journal of Clinical Pharmacy (IJCP). Throughout his time as editor-in-chief, he encouraged students and practitioners to publish their work. This journal, originally named Pharm. Weekblad – Scientific Edition, which was a scientific publication, became known first, as Pharmacy World & Science (PWS), and then in 2010, was renamed to IJCP. His work in this regard was transformative and leads to the remoulding of the journal into one with a substantial international impact.

Foppe was a great supporter of evidence-informed pharmaceutical policy at a global level. In 2009, he helped publish a key editorial in “Pharmacy World and Science” regarding the starting of the journal “Southern Med Review”, later renamed as “Journal of Pharmaceutical Policy and Practice”, as he believed there was a paucity of journals focused on pharmaceutical policy, which were needed to complement the more practice-based ones [25].

At a later stage of his career, he edited a book aimed at helping practitioners worldwide to implement pharmaceutical care. This was indeed the main aim of his life, to transform standard pharmacy practice, or usual care as it is often called in randomised controlled trials, into pharmaceutical care and make this advanced way of constantly optimising medication usual practice. In this book, he gathered more than 40 worldwide reputed authors and covered all aspects believed to be essential for practice implementation, from disease-specific to health care setting-specific, to country-specific and of course not forgetting about university education and continuous professional development [26].

He will be greatly missed by all his friends, colleagues and followers throughout the world.

## Data Availability

Not applicable

## Abbreviations

DMP: Disease management programme
DRPs: Drug-related problems
ESCP: European Society of Clinical Pharmacy
GP: General practitioner
FIP: International Pharmaceutical Federation
HRQoL: Health-related quality of life
Int J Clin Pharm: International Journal of Clinical Pharmacy
KNMP: Koninklijke Nederlandse Maatschappij ter bevordering der Pharmacie
PCNE: Pharmaceutical Care Network Europe
PEFR: Peak expiratory flow rate
PhC: Pharmaceutical care
WHO: World Health Organization
